# Ecological tips to reduce waste during acetic acid dye application for Barrett’s esophagus evaluation: a small syringe in the accessory channel is enough!

**DOI:** 10.1055/a-2139-3897

**Published:** 2023-08-23

**Authors:** Elena De Cristofaro, Raphaelle Grau, Pierre Lafeuille, Clara Yzet, Florian Rostain, Jérôme Rivory, Mathieu Pioche

**Affiliations:** 1 Department of Systems Medicine, Gastroenterology and Endoscopy Unit, University of Rome Tor Vergata, Rome, Italy; 2 Department of Endoscopy and HepatoGastroenterology, Pavillon L, Edouard Herriot Hospital, Lyon, France

It is common knowledge that endoscopic procedures have a non-negligible ecological impact 1 , and every effort to address this impact can contribute to reduction in the associated waste and ecological footprint of the procedure 2 .

We previously reported tips and tricks to reduce the waste in daily endoscopic practice, from simple procedures such as gastric sampling 3 , to more complex ones such as gastric peroral endoscopic myotomy or endoscopic submucosal dissection 4 5 .

Herein, we report the case of a patient with Barrett’s esophagus (BE), in whom a careful evaluation of the esophagus was required due to previous endoscopic submucosal dissection for dysplasia. Chromoendoscopy with acetic acid is commonly used in the evaluation of BE. Acetic acid is a reactive dye that is sprayed onto the BE, causing a reversible chemical reaction with the proteins in the cytoplasm, resulting in aceto-whitening of the Barrett’s mucosa and focal erythema; dysplastic areas are revealed after quicker loss of aceto-whitening compared with nondysplastic BE.

The acetic acid is usually applied to the esophageal mucosa either with a spray catheter and a 20 mL syringe or through a 60 mL syringe attached to the endoscope valve. The weight of these devices is almost equivalent (28 g vs. 31 g, respectively) ( Fig. 1 ). Although the 20 mL syringe usually requires the additional spray catheter, in the current case we used a 20 mL syringe applied directly to the accessory channel of the endoscope and used a common flushing adapter (Endogator; Steris Endoscopy, Mentor, Ohio, USA) ( Fig. 2 ). After evaluation using narrow-band imaging and optimal dyeing of BE with acetic acid, no dysplastic lesions were revealed ( Video 1 ).

**Fig. 1:**
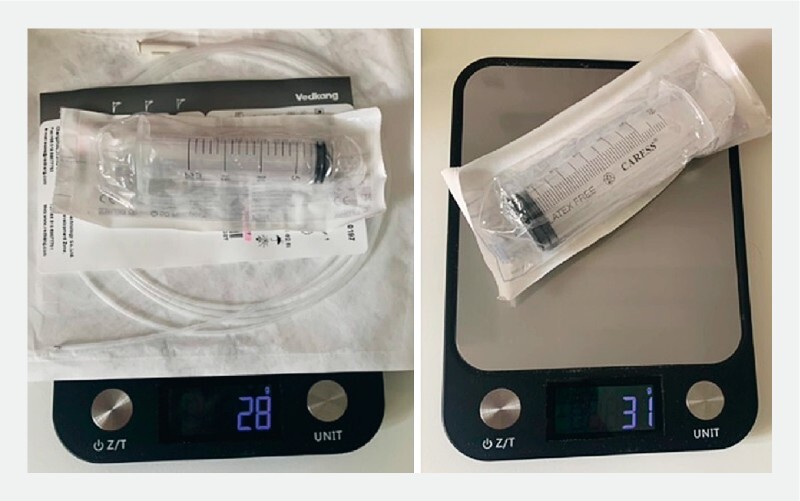
Weight of 20 mL syringe plus catheter spray and 60 mL syringe for the scope valve.

**Fig. 2:**
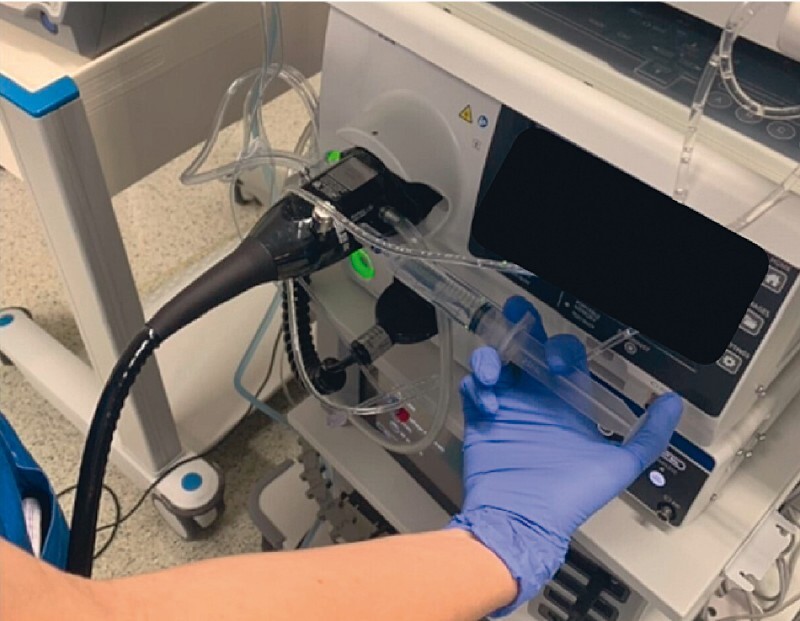
Application of acetic acid directly through the 20 mL syringe via the accessory channel of the endoscope using the common flushing adapter (Endogator; Steris Endoscopy, Mentor, Ohio, USA).

**Video 1** Ecological tips to reduce waste during acetic acid dye application for evaluation of Barrett’s esophagus.


From an ecological perspective, the use of this technique is an efficient and straightforward alternative, resulting in less plastic waste. It could also be applied during colonic and gastric chromoendoscopy. Every act counts and can help to preserve our planet.

Endoscopy_UCTN_Code_TTT_1AO_2AB

## Notes

Endoscopy E-Videos

https://eref.thieme.de/e-videos
*E-Videos* is an open access online section of the journal *Endoscopy* , reporting on interesting cases and new techniques in gastroenterological endoscopy. All papers include a high-quality video and are published with a Creative Commons CC-BY license. Endoscopy E-Videos qualify for HINARI discounts and waivers and eligibility is automatically checked during the submission process. We grant 100% waivers to articles whose corresponding authors are based in Group A countries and 50% waivers to those who are based in Group B countries as classified by Research4Life (see: https://www.research4life.org/access/eligibility/ ). This section has its own submission website at https://mc.manuscriptcentral.com/e-videos
